# Retraction: “Sudden transient blindness following ureteroscopy: an uncommon complication of a common procedure (a case report) (Pan African Medical Journal. 2024;47:201. doi: 10.11604/pamj.2024.47.201.42952)”

**DOI:** 10.11604/pamj.2024.48.167.43984

**Published:** 2024-08-09

**Authors:** The Pan African Medical Journal

**Affiliations:** 1 The Pan African Medical Journal, Park Suite Building, Parklands Road, Nairobi, Kenya

**Keywords:** Retraction, data fabrication, case report, PAMJ

## Abstract

This article retracts the published article “Sudden transient blindness following ureteroscopy: an uncommon complication of a common procedure (a case report)” Pan African Medical Journal. 2024;47: 201. doi: 10.11604/pamj.2024.47.201.42952 by Wael Gazzah, Sedki Masmoudi, Hamza Chakroun, Rayen Lahouar, Bacem Zaidi, Badreddine Ben Khalifa, Sahbi Naouar, Braiek Salem.

## Retraction

We, the editors of the Pan African Medical Journal would like to notify the PAMJ readers of the retraction of the article “Sudden transient blindness following ureteroscopy: an uncommon complication of a common procedure (a case report)” Pan African Medical Journal. 2024;47: 201. doi: 10.11604/pamj.2024.47.201.42952 by Wael Gazzah, Sedki Masmoudi, Hamza Chakroun, Rayen Lahouar, Bacem Zaidi, Badreddine Ben Khalifa, Sahbi Naouar, Braiek Salem, published on April 22, 2024 [1].

An internal investigation was carried out, concluding that there was sufficient evidence of data fabrication, publication without the patient's consent, and a wrong diagnosis by Wael Gazzah *et al*. The authors agreed with the accusations and approved the retraction. We therefore retract this article from the literature following guidelines and best editorial practices from the Committee on Publication Ethics. We apologize to our scientific community for this unfortunate situation.

The retracted article will remain online to maintain the scholarly record, but it will be digitally watermarked on each page as “Retracted.”
